# Pathogenesis of Drug Induced Non-Allergic Angioedema: A Review of Unusual Etiologies

**DOI:** 10.7759/cureus.1598

**Published:** 2017-08-23

**Authors:** Junior Kalambay, Haider Ghazanfar, Karen A Martes Pena, Ruhul A Munshi, George Zhang, Jay Y Patel

**Affiliations:** 1 Medicine, University Setif; 2 Internal Medicine, Newark Beth Israel Medical Center; 3 Internal Medicine, Chittagong Medical College & Hospital; 4 Internal Medicine, Shanghai Medical College, Fudan University; 5 Internal Medicine, Pramukhswami Medical College, Anand,Gujarat

**Keywords:** angioedema, hereditary angioedema, non-allergic, causality, drug hypersensitivity, drug induced reactions, urticarial, hypersensitivity, female

## Abstract

Angioedema is the swelling of mucosal and sub-mucosal tissue. Typically, it manifests as the swelling of the face, lips, and tongue. Angioedema can be severe and life threatening when it involves the respiratory tract. Drug induced allergic angioedema and drug induced non-allergic angioedema differ in their mediator, their clinical presentations, and their management. In drug induced non-allergic angioedema, symptoms are resistant to antihistamine and corticosteroid treatment. The aim of the analysis was to identify which medications are associated with drug-induced non-allergic angioedema and to understand the mechanism of action via which of these medication cause angioedema.

## Introduction and background

Angioedema is the swelling of mucosa and submucosal tissue. Typically, it manifests as the swelling of the face, lips, and tongue. Angioedema can be severe and life threatening when it involves the respiratory tract [1]. Drugs are second to food as the most common cause of angioedema cases seen in the emergency department [2]. Drugs may induce two different types of angioedema; allergic and non-allergic angioedema [3]. Studies have shown that to date, most physicians in the emergency department do not recognize the specific type of angioedema presenting in a patient, and they don’t treat the angioedema episode considering the difference in management that each type of angioedema implies [4]. Indeed, drug induced allergic angioedema and drug induced non-allergic angioedema differ in their mediator, their clinical presentations, and their management.

Drug induced allergic angioedema is a type I hypersensitivity and mediated by histamine [5]. In type I hypersensitivity, the medication cross link with immunoglobulin E (IgE) antibody bound on the surface of mast cells which results in the release of histamine [6]. Clinically, drug induced allergic angioedema will present with the rapid onset of swelling of mucosa and submucosa tissues. The patient will also have a typical urticarial rash. Symptoms will respond quickly to antihistamine, epinephrine and corticosteroid treatment [1].

Drug induced non-allergic angioedema is mediated by bradykinin [7]. In drug induced non-allergic angioedema the urticarial rash is absent. In addition, the onset is more progressive as compared to histamine mediated angioedema. Symptoms may subside in three to five days [7]. In drug induced non-allergic angioedema, symptoms are resistant to antihistamine and corticosteroid treatment, symptoms resolve only after drug discontinuation [8].

Because bradykinin mediated angioedema is under recognized, poorly managed, and the patients have an unfavorable outcome, this article will review drug induced non-allergic angioedema. The drugs covered in this article are selected as examples of key targets in the kallikrein-kiting system (KKS), and because together they cover the entire KKS from bradykinin synthesis to bradykinin receptors.

Article published on PubMed index journal between 1978 and 2017 and related to the topic of interest were included in the study. The keywords used to search the article included “angioedema”, “non-allergic”, “causality”,“ drug hypersensitivity”, “histamine antagonists”, “urticaria”, “bradykinin”, “epidemiology”, “drug combinations” and “gender”. A total of 138 articles were reviewed and analyzed. Out of 138 articles; 40 articles were found to be pertinent to our study and were included in the study.

## Review

The kallikrein-kinin cascade and its interactions with the renin-angiotensin-aldosterone and the complement systems

The KKS is a cascade of proteolytic enzymes that release vasoactive peptides. Plasma kallikrein cleaves human high molecular weight kininogen and releases bradykinin [9]. Bradykinin stimulates beta-2 adrenergic (B2) receptors which result in the release of nitric oxide and prostacyclin [10-11]. Nitric oxide and prostacyclin release result in local vasodilation and increased vascular permeability which leads to the development of angioedema [12]. The KKS antagonizes the renin angiotensin aldosterone system (RAAS) in its vascular effect [13]. The KKS and RAAS are coupled by the angiotensin converting enzyme (ACE). The ACE degrades bradykinin in the KKS and synthesizes Angiotensin II from Angiotensin I in the RAAS [14].

Another cascade of proteolytic enzymes is the complement system. Once activated, the complement system will produce anaphylatoxins (C3a, C4a, and C5a) and membrane attack complex. Anaphylatoxins and bradykinin have a similar mechanism of action. They increase local vascular permeability and cause vasodilation. The membrane attack complex also causes cell lysis [15-17].

**Figure 1:**
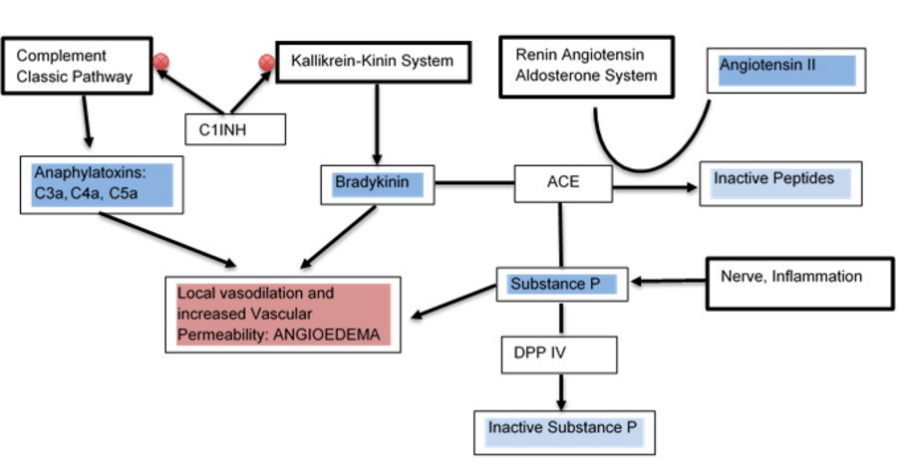
Interaction of kallikrein-kiting system (KKS), renin angiotensin aldosterone system (RAAS) and complement system in the development of angioedema

The KKS is also coupled to the complement system by the C1 Inhibitor (C1INH). The C1INH is a serine protease that inhibits the KKS via the inactivation of factor XIIa and kallikrein and inhibits the complement system via the inactivation of C1r and C1s of the classical pathway [18-20]. Interaction of KKS, RAAS and complement system in the development of angioedema has been illustrated in Figure 1.

Simvastatin and bradykinin type two receptors

A 75-year-old African American female patient with type 2 diabetes and dyslipidemia was admitted to the hospital with recurrent night episodes of facial, lip and tongue swelling. The patient denies any rash during these episodes and mentioned that self-medication with diphenhydramine did not relieve her symptoms. The patient was hemodynamically stable. The patient's normal level of C4, C1 esterase inhibitors, and C1q binding assay ruled out the diagnosis of hereditary angioedema. Her medications were reviewed, Simvastatin dose was increased each night from 10 to 20 mg, nine months prior to admission. During this period, episodes of angioedema woke the patient up almost each night. Simvastatin was considered as the possible trigger of these episodes of angioedema and was discontinued. Oxygen saturation was monitored continuously. The patient facial, lip and tongue swelling resolved over the next 36 hours without the use of any further corticosteroid treatment. In the following six months, the patient reported only one episode of daytime angioedema but no new episode of nighttime angioedema [21]. In this case report, bradykinin is most likely the mediator of angioedema episode because of at least four facts; there was no urticarial rash, the relatively long delay of angioedema onset after the simvastatin administration, the lack of improvement of the swelling with diphenhydramine, and the resolution of angioedema after simvastatin discontinuation.

At least two mechanisms have been identified in Statins induced non-allergic angioedema. Firstly, statins increase expression of bradykinin type 2 receptors on endothelial cells. In an experience where human coronary endothelial cells were cultured, Lovastatin upregulated bradykinin type two receptors. Secondly, statins may also potentiate the action of bradykinin on its receptors. Both mechanisms can make a patient susceptible to develop angioedema with circulating level of bradykinin through the increased release of prostacyclin and nitric oxide [12, 22].

Sitagliptin, angiotensin converting enzyme and dipeptidyl peptidase IV

A case reported concluded that Sitagliptin is associated with drug induced non-allergic angioedema. In this case report a 79-year old female with type 2 diabetes previously taking detemir insulin, atorvastatin 20 mg, and Irbesartan 150 mg was started on sitagliptin due to the high glycated hemoglobin (HbA1c) which was 8.1%. The patient developed angioedema with swelling of her lips, tongue, and mouth 14 days after starting sitagliptin. Sitagliptin was discontinued and remission was observed within a few days without supportive medication. Ten days later, the sitagliptin was re-started. The patient developed angioedema again two days later. Sitagliptin was discontinued permanently. This case describes the scenario of an oral drug challenge to confirm non-allergic angioedema: the urticarial rash is not present, symptoms are only induced by the administration of sitagliptin and they have a delayed onset. The discontinuation of the drug led to resolution of all symptoms [23].

Sitagliptin is a dipeptidyl peptidase IV (DPP-IV) inhibitor. The DPP-IV inactivates the incretins glucose-dependent insulinotropic polypeptide and glucagon-like peptide [24]. As a result, DPP-IV improves glycemic control in type 2 diabetes mellitus [25]. In addition to incretins, DPP-IV also inactivates substance P, a vasoactive peptide that increases vascular permeability by its action on neurokinin receptor 1 (NK1) [26]. Substance P is involved in ACE inhibitor (ACEI) associated angioedema. Studies have demonstrated that Substance P levels are increased during ACEI associated angioedema [27]. Also, infusion of bradykinin or substance P caused tracheal edema in the rate [28]. Both bradykinin and substance P are degraded by ACE. During ACE inhibition, accumulation of Substance P doesn’t cause angioedema because it is inactivated by DPP-IV. Thus, the addition of DPP-IV inhibitor such as Sitagliptin may cause angioedema in susceptible patients [29-30].

Vildagliptin, another DPP-IV inhibitor has not been associated with angioedema when it is taken alone. It has been shown to causes angioedema in the patients who are also taking ACEI concomitantly [31]. This latter finding confirms the contribution of substance P in the pathogenesis of angioedema.

Risperidone and C1 inhibitor

To date, only four cases of risperidone have been reported to be associated with angioedema [32-35]. Among them, a case-report described an episode of angioedema that occurred in a 30-year-old female treated for the schizoaffective disorder. The patient developed facial and periorbital edema without urticaria two weeks after risperidone was started at 6 mg dose. When the dose was halved, symptoms subsided, but the mental status deteriorated. Risperidone was then increased to 6 mg/day but resulted in the recurrence of the facial and periorbital edema. Risperidone was stopped and the facial and periorbital edema resolved completely over two weeks. The Immunology laboratory test results showed a normal level of C3 and low levels of C4 and C1 esterase inhibitor [34]. A similar case of angioedema was reported in a 38-year-old female patient with amphetamine induced mood disorder who was admitted for aggressiveness, irritability, and grandiose thoughts. Risperidone was started at 2 mg and then increased to 6 mg/day. Nine days after the treatment, the patient developed facial and periorbital swelling. Laboratory showed normal levels of C3, C4, and C1 esterase inhibitor. Risperidone was discontinued; low dose hydrocortisone and hydroxyzine were prescribed. The swelling resolved completely within four days. The clinical pattern of angioedema episodes in these cases supports a non-allergic form of angioedema because there was no urticaria. In addition, the delayed onset of symptoms after medication was administrated, and the progressive recovery is less likely to be the manifestation of a type I hypersensitivity reaction.

The chronology of symptoms in both cases is more consistent with bradykinin-mediated angioedema. Finally, the complete resolution of symptoms after the discontinuation of risperidone supports bradykinin-mediated angioedema. Risperidone causes non-allergic angioedema by two mechanisms. The first mechanism is the suppression of C1INH [34]. This is supported by the low level of C1INH, normal level of C3 and low-level C4 found during these two case reports. Normally, C1INH inhibits the complement system and the KKS. The C1INH suppression will increase spontaneous activation of the classical pathway of the complement and anaphylatoxins production (C3a, C4a, C5a) [18]. Anaphylatoxins will cause vascular permeability and vasodilation [16]. The C1INH inhibition will also leave the KKS unopposed with increased synthesis of bradykinin. Both actions will result in angioedema. The second mechanism is the aggregation of bradykinin. In this case, the patient will have a normal level of C1INH, C3, and C4 (32). Bradykinin triggers the release of nitric oxide and prostacyclin by endothelial cells. Nitric Oxide and prostacyclin will increase vascular permeability and will lead to the development of angioedema [12].

Summary of mechanism of action of drug induced non-allergic angioedema

To our knowledge, drugs can target three different sites of actions in the KKS to cause bradykinin mediated angioedema:

1. The synthesis of bradykinin with C1 INH suppression: Risperidone increases bradykinin production by inhibiting the C1 INH. The same mechanism also increases complement activity

2. The response to bradykinin: Bradykinin receptors type 2: Statins for example increase blood gene BR2 expression and their sensitivity to bradykinin

3. The metabolism of bradykinin and substance P with ACE and DPP-IV suppression: The action of ACEI on ACE and Sitagliptin on DPP-IV inhibits Substance P degradation and inactivation respectively. In addition, ACEI also decreases the metabolism of bradykinin

Management

Drug induced non-allergic angioedema (bradykinin mediated angioedema) will present with some characteristic features in the clinical presentation. It is important to distinguish non-allergic from allergic angioedema in order to treat the patient accordingly. The speed of onset is characteristic. Histamine mediated angioedema starts less than an hour after the exposure and bradykinin mediated angioedema may take several hours to days to develop. Urticaria is never present in bradykinin mediated angioedema. Abdominal manifestations are more common in drug induced non-allergic angioedema than in drug induced allergic angioedema [36].

Respiratory tract involvement is uncommon in bradykinin mediated angioedema. The most important action to take when a drug induced non-allergic angioedema is suspected is to discontinue the offending drug [7-8]. In Histamine induced angioedema, symptoms resolve within 24 hours. Symptoms last longer in bradykinin mediated angioedema and can last up to five days [37]. Antihistamine, epinephrine, and corticosteroid have not shown to decrease the duration of symptoms [38]. Supportive care is provided if the respiratory tract is involved.

Although laboratory results are not available early enough to guide the treatment, it is important to measure C4 level and tryptase level to rule out other etiologies and to ensure proper follow-up. The C4 level is always decreased in hereditary angioedema. The diagnosis of hereditary angioedema is more likely in the absence of obvious etiology. Tryptase is elevated in allergic angioedema [37-38]. The patient who does not have any involvement of the tongue, larynx or any other airway compromise can be discharged after 12 to 24 hours of observation.

A patient who had life threatening episode of angioedema should discontinue the medication indefinitely. the blood results can be evaluated in an outpatient setting with the primary care physician. If hereditary angioedema (HAE) is suspected, the patient should be referred to an immunologist for further evaluation and prophylactic treatment [39-40].

## Conclusions

Drug induced angioedema is a common scenario in the emergency department. It can be a life threatening when airways are involved. Many physicians approached it like an allergic reaction. There is a need to increase awareness about drug induced non-allergic angioedema as its management is different from allergic angioedema. In the case of the nonallergic reaction, stopping the offending agent is the most important action to take.
